# S131. PERCEPTUAL ABNORMALITIES AND RELIGIOSITY IN ULTRA HIGH-RISK FOR PSYCHOSIS (UHR) INDIVIDUALS IN A LATIN AMERICAN POPULATIONAL SAMPLE RESULTS FROM THE SAO PAULO SSAPP COHORT

**DOI:** 10.1093/schbul/sby018.918

**Published:** 2018-04-01

**Authors:** Alexandre Loch, Camille Chianca, Elder Lanzani Freitas, Julio Cesar Andrade, Tania Maria Alves, Mauricio Henriques Serpa, Lucas Hortêncio, Martinus Theodorus van de Bilt, Wagner Gattaz, Wulf Rössler

**Affiliations:** 1 University of Sao Paulo; 3 Charité, University of Zurich, ZInEP, University of Sao Paulo

## Abstract

**Background:**

In the last decades, the ultra-high risk for psychosis (UHR) status has been studied to prevent people from developing full-blown psychosis. To better understand this condition, and to better predict conversion, the relationship between UHR and several biological and environmental markers have been assessed. Nevertheless, most UHR studies come from developed countries, constituting a gap in the literature regarding culture-related environmental factors. This study aims to study religiosity, a peculiar cultural constituent of Latin-American societies, in populational samples of UHR individuals and controls from the city of Sao Paulo, Brazil.

**Methods:**

These are partial results from the cohort project SSAPP (Subclinical Symptoms and Prodromal Psychosis).

Over 2500 individuals aged between 18 and 30 years were asked to participate in the research in a household survey. The Prodromal Questionnaire was used, a screening instrument for UHR constituted of 92 yes-or-no items divided into 4 domains; positive, negative, disorganization, and general symptoms. Those with 18 points or more in the positive subscale were asked to come to the Institute of Psychiatry to undergo blood testing, neuropsychological evaluation, to complete several self-filling questionnaires, and to undergo a clinical interview with an experienced psychiatrist with the SIPS (Structured Interview for Prodromal Syndromes). Functioning was assessed with the Global Assessment of Functioning (GAF), and religiosity was evaluated with the DUREL (Duke religiosity Index), which measures organizational and non-organizational religious activity, and intrinsic religiosity.

Total sample for this study was constituted of 60 UHR individuals and 91 controls.

The scores on the 5 positive symptoms items in the SIPS, the three religiosity dimensions, and the GAF were correlated, in controls and in UHR individuals.

All variables had a non-normal distribution (Kruskal-Wallis p<0.001), so Spearman’s test was used. Generalized linear model was used between the resulting significantly associated variables.

SPSS 23 for Mac was used for the analysis.

**Results:**

Level of organizational and non-organizational religious activity did not differ between samples, but controls had significantly higher levels of intrinsic religiosity than UHR individuals (p=0.04).

None of the religiosity measures were related to positive symptom items for controls. For UHR individuals, P4 (perceptual abnormalities) was positively related to organizational religiosity (p=0.001).

GAF was not related to any P item in controls, but they inversely correlated to P4 (perceptual abnormalities, p=0.033) and P5 (disorganized communication, p=0.031) in UHR individuals. GAF was not correlated to religiosity, neither in UHR nor in controls.

Generalized linear model using P4 as dependent variable and GAF and Organizational religiosity as independent variable showed that organizational religiosity determined P4 score rather than GAF.

**Discussion:**

Results indicate that a higher score on perceptual abnormalities was related to a higher attendance in churches/temples in UHR individuals in our sample. Our study sheds light to an important aspect of Latin American cultures, namely the relationship between exceptional experiences and religion in lay people. In developing countries with a lack of mental health services churches might act as important gatekeepers for UHR individuals. Since an expected inverted correlation between GAF and religiosity was not found, we might hypothesize that religion might have been used to cope with subclinical symptoms. Longitudinal data would be required to test this hypothesis.

